# Epigenetics and reproductive isolation: a commentary on Westram et al., 2022

**DOI:** 10.1111/jeb.14033

**Published:** 2022-09-05

**Authors:** Nicholas P. Planidin, Clarissa F. de Carvalho, Jeffrey L. Feder, Zachariah Gompert, Patrik Nosil

**Affiliations:** ^1^ CEFE, Univ Montpellier, CNRS, EPHE, IRD Montpellier France; ^2^ Departamento de Ecologia e Biologia Evolutiva UNIFESP Diadema Brazil; ^3^ Department of Biological Sciences University of Notre Dame Notre Dame Indiana USA; ^4^ Department of Biology Utah State University Logan Utah USA

## 1 INTRODUCTION

Reproductive isolation (RI) is often considered an essential component of speciation; however, its definition varies, and it is challenging to measure, making it difficult to compare across studies. To help overcome these difficulties, Westram et al. (2022) suggest to define RI as ‘a quantitative measure of the effect of genetic differences on gene flow’, specifically at neutral loci. Here, we consider this definition of RI in the context of epigenetic variation. We define epigenetics as molecular interactions with DNA that influence gene expression without changes in the underlying nucleotide sequence (see Box 1 for background on epigenetic variation). Furthermore, we consider a process as epigenetic, regardless of whether it is transmitted across generations or not. As we will see, intergenerational transmission of epigenetic state is not a prerequisite to influence RI. In the following, we establish a framework through which to better quantify epigenetic influences on population divergence and speciation by building upon the model of RI at neutral loci established by Westram et al. (2022).

BOX 1 **Epigenetics in natural populations**

**FIGURE B1:**
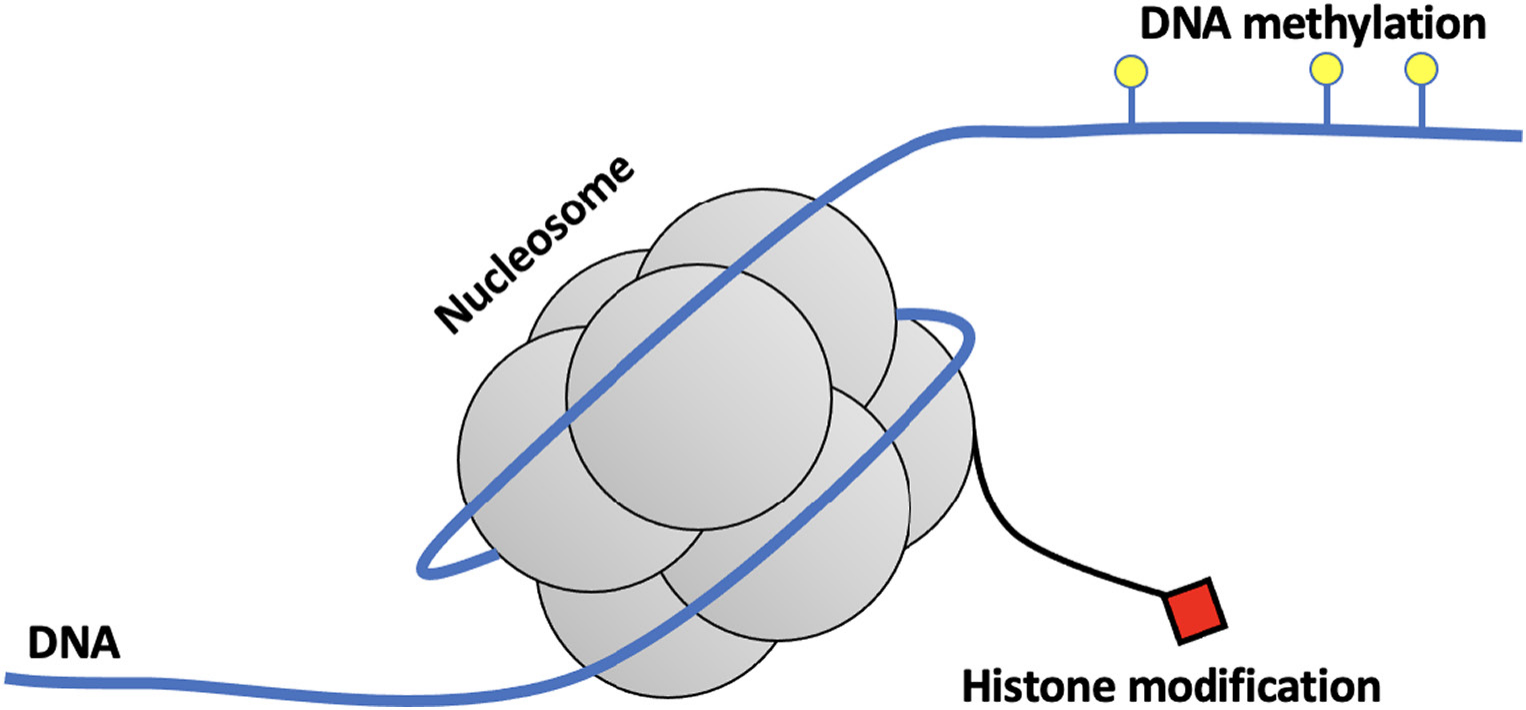
Schematic of DNA methylation and histone modification

Two examples of epigenetic marks are DNA methylation, in which a methyl group is covalently bonded by a cytosine to form methyl‐cytosine, and histone modifications, in which the tail of a histone (a component of DNA packaging nucleosomes) undergoes one of many forms of modification, for example, methylation, acetylation or phosphorylation (Figure B1). These tags can alter processes such as DNA packaging and gene expression, by influencing the action of DNA interacting proteins such as histones and transcription factors (Gibney & Nolan, 2010). These epigenetic marks are phylogenetically widespread (Feng & Jacobsen, 2011; Glastad et al., 2019) and can influence adaptive phenotypes (Baerwald et al., 2016; Rangani et al., 2012). Among epigenetic marks, DNA methylation is the best understood and most widely studied, particularly in the context of natural populations (Hu & Barrett, 2017; Kilvitis et al., 2014; Vandegehuchte & Janssen, 2014). DNA methylation state can be passed from parent to offspring (Yagound et al., 2020) and differs among populations in divergent environments (Dubin et al., 2015; Wogan et al., 2020; for examples of both see Heckwolf et al., 2020; Verhoeven & Preite, 2014).

Epigenetics can underlie phenotypic plasticity. A large body of work has examined phenotypic plasticity (West‐Eberhard, 2003), including its effects on gene flow and speciation (Fitzpatrick, 2012; Klemetsen, 2010; Otte et al., 2016; Thibert‐Plante & Hendry, 2011). However, focussed studies that have directly examined epigenetic variation as a mechanism producing barriers to reproduction (Lafon‐Placette & Köhler, 2015; Smith et al., 2016) and speciation (Greenspoon et al., 2022) are only now emerging.

## 2 EPIGENETICS AND RI: A CONCEPTUAL FRAMEWORK

**FIGURE 1:**
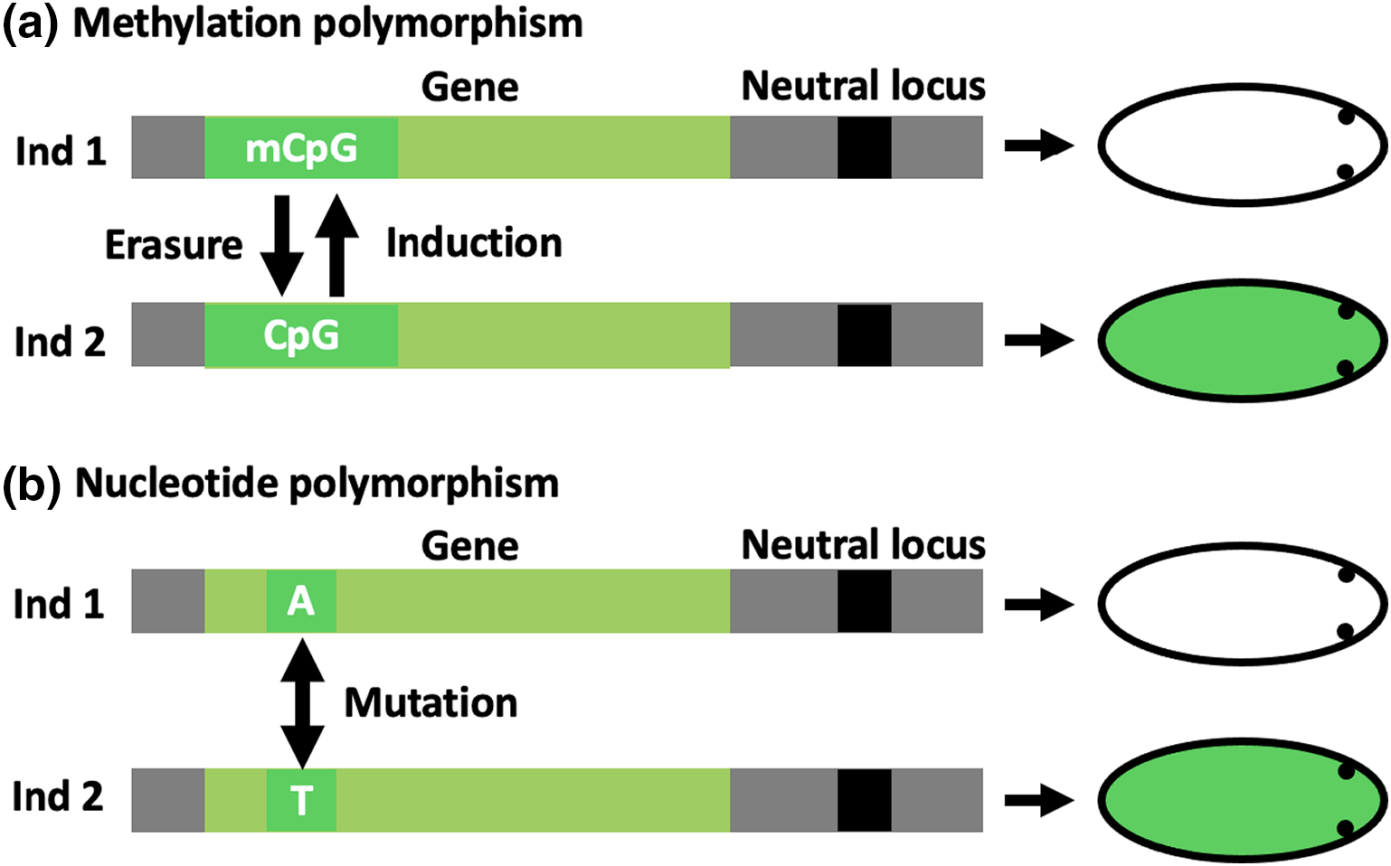
Example of how an epigenetic locus (CpG) and genetic locus (A/T) can produce a phenotype and interact with a linked neutral locus

In their examination of RI, Westram et al. (2022) focus on two genetic loci, a locus under divergent selection between two environments and a neutral locus linked with the locus under selection. In an analogous fashion, we consider an epigenetic locus and a neutral genetic locus linked to it. A well‐studied example of an epigenetic locus is the cytosine‐guanine dinucleotide (CpG), which can be methylated to form mCpG (Feng et al., 2010; Zemach et al., 2010). One mechanism through which methylation state can change is in response to an external signal from the environment, and such change in methylation state can alter the phenotype of an individual without a change in nucleotide sequence (Angers et al., 2010; Schmid et al., 2018). We shall call this process *induction*. This induction of the methylated state can also have a variable degree of stability over time, being lost during the life of an individual, or during epigenetic remodelling in germ cells and early embryos of some organisms (Feng et al., 2010; Santos & Dean, 2004). We shall call this process *erasure*. We can, therefore, imagine an epigenetic locus whose state (in this example methylated or unmethylated) encodes a phenotype and a neutral genetic locus that is linked with such epigenetic locus (Figure 1a), similar to a genetic locus (Figure 1b). Consequently, we consider variation at the epigenetic locus being produced by induction and erasure, rather than mutation (Figure 1). As we will see, these differences can have consequences for RI.

When two habitats provide strong divergent environmental induction, two populations can form distinct epigenetic‐dependent phenotypes, which produce RI. If the epigenetic marks are completely erased between generations, the state of the epigenetic locus only effects the fitness of the migrants between habitats and not their offspring, thus producing a uniform and *global* (genome‐wide) reduction in gene flow to all neutral loci across the genome. In this context, epigenetic variation will act like a geographic barrier, as described by Westram et al. (2022). In contrast, if the epigenetic mark is not erased between generations, there will be additional selection against recombination surrounding the epigenetic locus. This will cause a *local* (restricted to parts of chromosomes) reduction in gene flow, influencing neutral loci dependent on their linkage with the epigenetic locus.

## 3 EXPANDING MODELS OF NEUTRAL GENE FLOW TO INCORPORATE EPIGENETICS

To highlight key similarities and differences between the effects of purely environmentally induced epigenetic versus genetically based barriers to neutral gene flow, we analyse the two‐population model with divergent selection acting on a single target locus, as presented by Westram et al. (2022). Epigenetic state is usually mediated by genetic variation to some extent (Adrian‐Kalchhauser et al., 2020), suggesting that much observed epigenetic variation may be due to underlying genetic variation. However, here we focus on epigenetic mechanisms independent of genetic sequence, as it is the simplest starting point and best corresponds to the framework of Westram et al. (2022).

For a two‐population, divergent selection model, Westram et al. (2022) define reproductive isolation (RI) as:

(1) $$\mathrm{RI}=1-\frac{m_{e}}{m}$$

where *m* is the gross migration rate or proportion of immigrants in a focal population after migration and *m*_e_ is the effective migration rate, representing the rate of migration which would have the same evolutionary effect for the introgression of a neutral allele into a population with no genetic barrier, as the actual migration rate *m* has in the population with a barrier (Bengtsson, 1985). In essence, selection and genetic barriers to gene flow will cause *m*_e_ to be less than *m*.

Given that *m* will be the same regardless of the underlying basis for selection, we compare estimates of *m*_e_ for epigenetic versus genetically based differences, to examine them as barriers to neutral gene flow. Building on earlier work of Bengtsson (1985), Charlesworth et al. (1997) derived an analytic approximation for *m*_e_ for a neutral allele (*n*) linked at varying recombination distances (*r*) to a target locus experiencing divergent selection (*s*) in a two‐population model, with selfing, which we modify here to consider only selection whereby:

(2) $$m_{e}\approx m\left(q+p\frac{\left(1-s\right)r}{1-\left(1-\mathrm{hs}\right)\left(1-r\right)}\right)$$

Equation 2 approximates *m*_e_ for the introgression of a neutral allele *n* from one population (population A) to another (population B), when selection follows migration. Selection acts symmetrically between populations on two alleles, *a* and *b*, segregating at a target locus X, with genotypes *aa*, *ab* and *bb*, having relative fitnesses 1, 1 − *hs* and 1 − *s*, respectively, in population A and the reverse in population B, where *h* is the dominance coefficient. It is assumed that *s* >> *m* and that *m* is relatively low. As a result, *p*, the frequency of the favoured allele *a* in population A at equilibrium prior to migration, is high, and *q*, the frequency of the disfavoured allele *b*, is low (~*m*/*s*). Thus, *m*_e_ in Equation 2 may be conceptualized as the extent to which neutral allele *n* carried by emigrant chromosomes from population A become disassociated by recombination with the disfavoured *a* alleles and introgress into the genetic background of population B before they are lost by selection acting on target locus X.

We now consider the effects that an epigenetic difference for target locus X would have for neutral gene flow. In this case, an epigenetic modification is environmentally induced in locus X in population A that is favoured locally but disfavoured in population B. One important factor is that unlike Mendelian inherited variation, the fidelity of intergenerational transmission (*v*) from parent to offspring of an epigenetic modification to a target locus is often low. Thus, in addition to recombination, reduced transmission will also dissociate neutral alleles *n* from the disfavoured epigenetically modified *a* state for the target locus X in the non‐inducing environment of population B, further facilitating the introgression of *n* alleles and increasing *m*_e_. To account for this:

(3) $$r_{v}=r+\left(1-r\right)\left(1-v\right)$$

**FIGURE 2:**
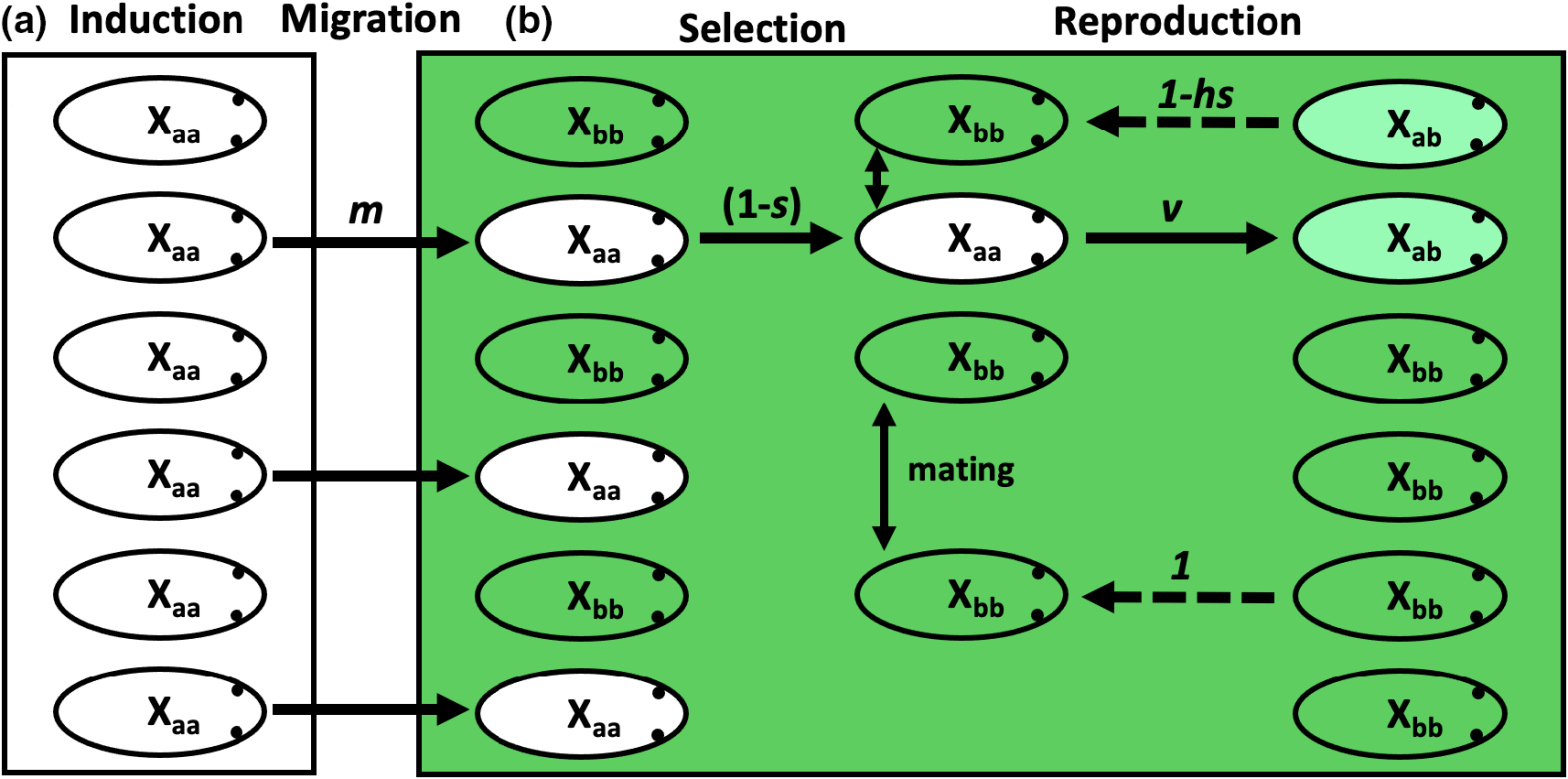
Visual representation of our two‐population, single epigenetic locus model. Individuals in habitat A have the epigenetic locus X induced to state X_aa_, producing the white phenotype. There is migration at rate *m* from habitat A to B, wherein white X_aa_ individuals have fitness 1 − *s* relative to green X_bb_ individuals. White X_aa_ individuals then hybridize with green X_bb_ individuals, passing the epigenetic state *a* onto hybrid X_ab_ offspring at rate *v*. Note, there are no crosses between X_aa_ migrants, since we assume *m* is small. Subsequent generations of X_ab_ hybrids then undergo cycles of selection and reproduction, with relative fitness 1 − *hs*, as indicated by the dashed arrows

*r*_*v*_ can be substituted for *r* in Equation 2, with $\left(1-r\right)\left(1-v\right)$ representing instances in which recombination does not disassociate the neutral allele *n* from the disfavoured epigenetic allele, but rather failure to transmit this allele does (Figure 2).

## 4 REPERCUSSIONS OF EPIGENETICS ON RI

**FIGURE 3:**
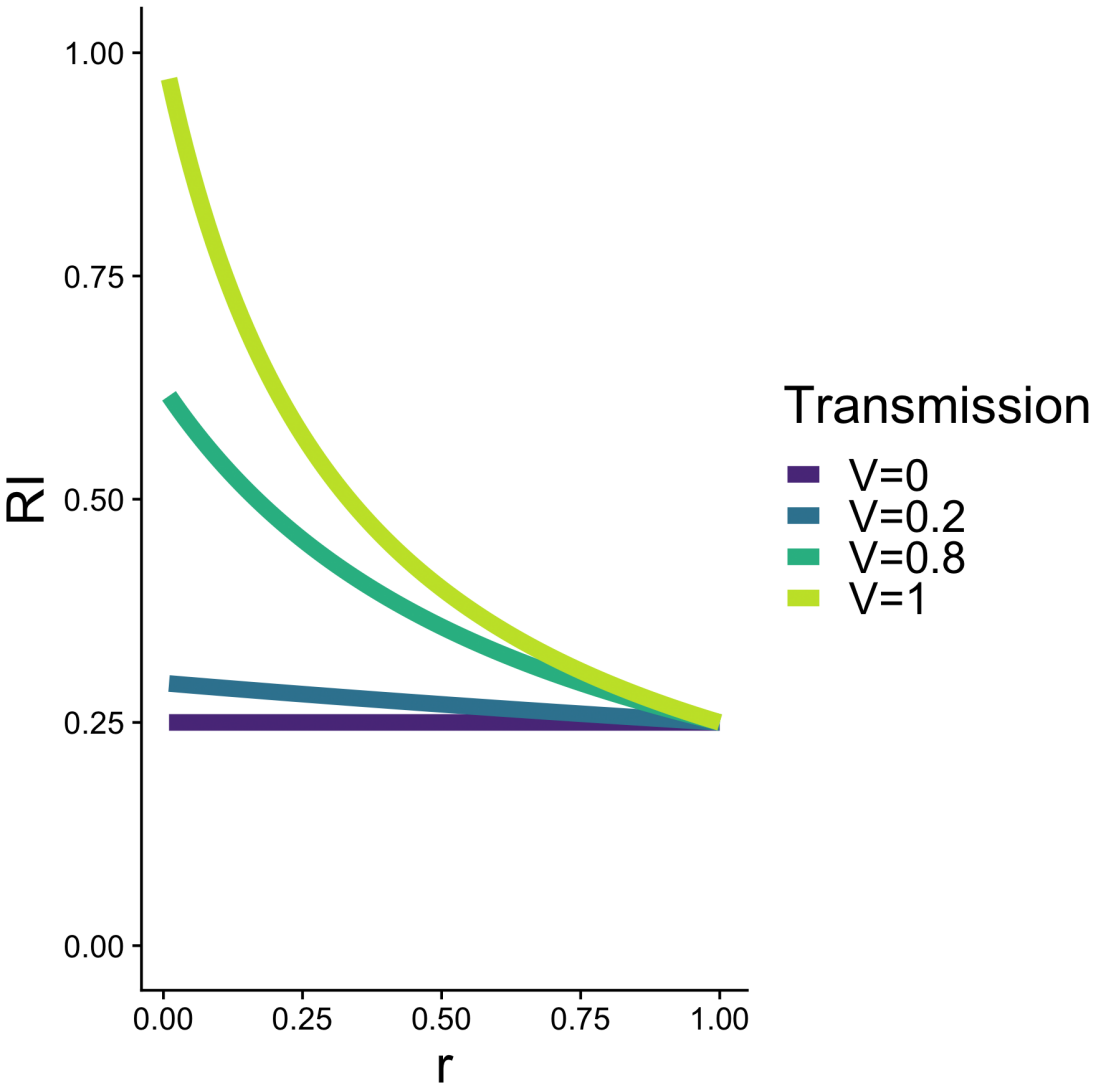
Reproductive isolation (RI) at a neutral locus with respect to recombination rate *r* with and epigenetic locus under divergent selection between two habitats, under different rates of intergenerational transmission (*v*) of epigenetic state. s = 0.25, m = 0.001, p = 1, q = 0

The key feature which dictates the effect of epigenetics on RI is transmission (*v*). When *v* = 0, there is no transmission of epigenetic state to the next generation; thus, *r*_v_ = 1, and *m*_e_ consequently reduces to *q* + *p*(1 − *s*). Under such circumstances, the epigenetic barrier to neutral gene flow is entirely due to selection acting against the individuals migrating from population A into population B. As recombination is not involved, the proximity of the neutral allele *n* to the target locus does not bear on the strength of the epigenetic barrier (Figure 3). Selection would, thus, be acting on the genome as a whole and have a uniform, genome‐wide effect on reducing neutral gene flow, analogous to geographic isolation as described by Westram et al., 2022), yet it is determined by the epigenetic state of the organism. In contrast, when *v* = 1, there is perfect intergenerational transmission of epigenetic state; thus, *r*_v_ = *r*, and estimates of *m*_e_ for epigenetic and genetic differences are the same. In other words, a perfect transmission of an epigenetic modification in population B is equivalent to Mendelian inherence of genetically based variation (Figure 3). Between these two extremes, epigenetic marks may exhibit a range of transmissibility and span from geographic‐like to genetic‐like effects on RI (Figure 3). Thus, there may be instances of increased local RI centred around sites lacking genetic variation, though they will be weaker than those due to genetic polymorphisms.

Whether epigenetic variation as considered in our model should be considered as a contributor to RI is an open issue. As described by Westram et al., RI must be based on genetic differentiation. Following this definition, an environmentally induced epigenetic state cannot produce RI since there is no genetic differentiation between populations. Thus, the reduction in gene flow due to selection directly against the epigenetic state of migrants should not be considered to reduce *m*_e_, despite such epigenetic differences having the same effect (1 − *s* in Equation 2) as a genetic difference for reducing gene flow in the parental migrant generation. Moreover, when the environmentally induced epigenetic state is even weakly transmissible, there will be a local reduction in gene flow around the epigenetic locus despite a lack of genetic differentiation, which may be interpreted as evidence of RI in empirical observations. In such circumstances, selection on the migrant generation due to an epigenetic difference would be included in the estimation of *m*_e_ and associated with RI akin to a genetic difference. We leave it to the reader to decide their position on this question, as it underscores the difficulty in conceptually defining RI.

While our model suggests that RI produced by epigenetic loci will generally be weaker than that produced by genetic loci, epigenetic barriers to gene flow are not necessarily negligible. The rapid loss of maladaptive epigenetic states due to low transmissibility (*v* < 1) means that, with strong gene flow, populations can be more divergent at epigenetic loci than equivalent genetic loci (q and p in Equation 2), producing greater interpopulation differences for selection to act on. There are observations of greater epigenetic than genetic differentiation among locally adapted populations (Dubin et al., 2015; Foust et al., 2016; Gugger et al., 2016; Herrera & Bazaga, 2016; Heckwolf et al., 2020; Johnson & Kelly, 2020; Lira‐Medeiros et al., 2010; Platt et al., 2015; Richards et al., 2012; Wogan et al., 2020; for counterexamples see Herden et al., 2019; Keller et al., 2016; Robertson et al., 2017). The major caveat to these observations is that epigenetic differentiation at many loci may be caused by differentiation at genetic loci. Furthermore, large epigenetic divergence does not necessarily underly environmentally induced and locally adaptive phenotypes.

There is much room to build additional nuance on top of this modelling framework. Here, we have presented an epigenetic model that is independent of genetic variation; however, as we emphasized above, genetic and epigenetic variation may not be independent. As explored by Greenspoon et al. (2022), epialleles may either promote speciation in accordance with genetic loci, or diminish speciation if they overwhelm genetic differentiation as the primary adaptive mechanism (see Table S1 for suggestions of how to incorporate further model complexity into the study of epigenetic RI).

## 5 INTEGRATING THEORY WITH DATA

If there is any influence of epigenetics on gene flow, it is not straight forward to make inferences about RI by measuring divergence between populations at neutral loci. As per the definition of RI proposed by Westram et al. (2022), the measurement of RI by introgression of neutral loci among populations attributes all RI to genetic differences. Yet, some RI may be due to unobserved epigenetic mechanisms. What must we measure to discern genetic from epigenetic RI? An important first step in understanding the role of epigenetics in RI is to observe the extent of epigenetic variation among natural populations (for example Gugger et al., 2016; Venney et al., 2021; Wogan et al., 2020). Then, it is necessary to establish if epigenetic differences between populations contribute to RI. The contribution of epigenetic state to RI can be inferred from the degree to which epigenetic state covaries with locally adaptive phenotypes (Baerwald et al., 2016; Kooke et al., 2015; Schmid et al., 2018), pre‐zygotic reproductive barriers (Smith et al., 2016) or post‐zygotic reproductive barriers (Lafon‐Placette & Köhler, 2015). However, correlating epigenetic differences to such traits is inherently limited, as it measures RI due to only one barrier to gene flow.

To measure epigenetic RI, we must estimate the transmission (*v*) of epigenetic marks in natural populations. Heckwolf et al. (2020) examined differences in DNA methylation among salt‐ and freshwater‐adapted populations of *Gasterosteus aculeatus*. They crossed individuals from the two habitats and exposed these mixed lineages to one of the divergent habitats for either one or two generations. Then, they showed that differentially methylated sites are transmissible (*v* > 0), as F2 crosses whose parents were exposed to the same divergent habitat, had DNA methylation profiles that were more like the natural population in that habitat, than those whose parents were not. Furthermore, epigenetic transmission can be estimated by directly comparing the epigenetic profile of parents and their offspring (Weyrich et al., 2016; Yagound et al., 2020); however, such observations do not specifically measure the transmission of locally adapted epigenetic marks. The primary caveat to such study designs is that the observed *v* is not precisely the same as the *v* in our model, as it is not independent of genetic variation. To more directly study the environmental induction of epigenetic marks, manipulative experiments can be performed with methyltransferase inhibitors (Biergans et al., 2012, 2015; Bossdorf et al., 2010; Herden et al., 2019; Wilschut et al., 2016), RNA interference (Bewick et al., 2019) and CRISPR‐Cas9 modification of epigenetic marks (Kang et al., 2019; Vojta et al., 2016). While these manipulative experiments give much greater power to observe the effects of epigenetic change in the absence of genetic change, they are difficult to implement in the natural setting.

**FIGURE 4:**
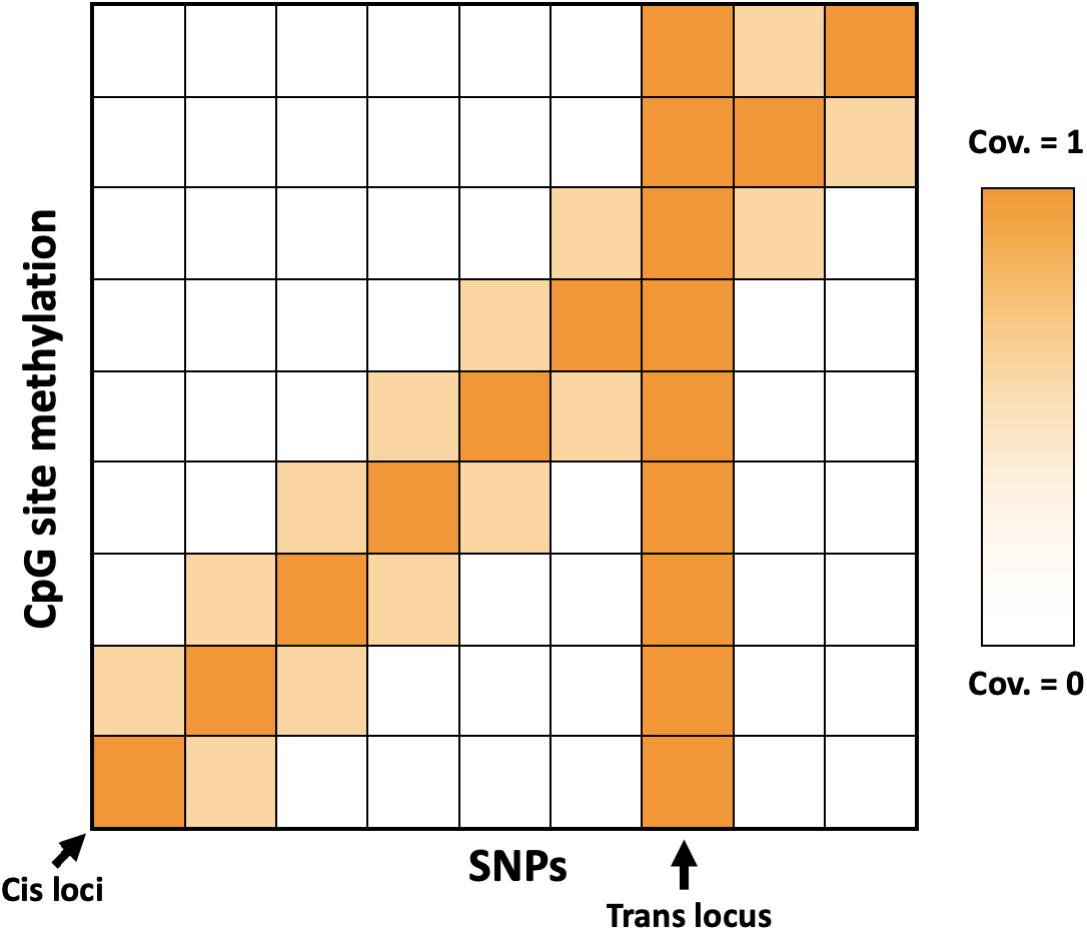
Example of a covariation matrix between methylation state at CpG dinucleotides and single‐nucleotide polymorphisms (SNPs). The orange tiles along the diagonal indicate interactions between SNPs and CpG sites adjacent to one another, whereas the vertical column of orange tiles indicates a single trans‐acting locus influencing the state of many CpG sites. Following the approach by Dubin et al. (2015)

We must investigate the genetic basis of epigenetic state, to understand the interplay between the two and its effect on RI. There are approaches to statistically control for genetic and geographic structure when testing for epigenetic differentiation among populations (Herrera et al., 2016; Lea et al., 2015, 2017); however, they typically apply a genome‐wide correction for relatedness, which does not capture locus‐specific interaction between genetic and epigenetic variation. To capture locus‐specific covariation between genetic structure and epigenetic state, one can perform a genome‐wide association study on epigenetic state, that is, treating epigenetic state as a molecular phenotype (Figure 4). Dubin et al. (2015) conducted such an analysis among accessions of *Arabidopsis thaliana*, allowing them to determine the extent to which cis‐ and trans‐acting genetic loci influence geographic variation in DNA methylation. To this end, Dubin et al. (2015) found that variation in methylation at single cytosines is roughly equally influenced by cis‐ and trans‐acting SNPs, whereas per cent gene body methylation is largely determined by trans‐acting SNPs. Notably, while it is useful to study asexual species to control for genetic effects on epigenetic state (Verhoeven & Preite, 2014), we must understand the genetic basis of epigenetic variation in sexually reproducing species to study its influence on RI.

Ideally, the observations which we have outlined could be made within a single‐model system. One would have to observe epigenetic variation in natural populations, assess the transmissibility of locally adaptive epigenetic states and parse the influence of genetic sequence and environmental induction on such states. After this, one can finally measure neutral gene flow with respect to these divergent epigenetic loci, to derive an estimate of epigenetic RI as per Equation (1. While implementing such an analysis is challenging, the studies highlighted above show that we can begin to make progress towards this ideal.

## 6 CONCLUSION

We have illustrated using a simple scenario, how epigenetics can influence neutral gene flow. However, it is becoming more apparent that epigenetic and genetic adaptation are inter‐dependent processes, rather than one being an epiphenomenon over the other (Danchin et al., 2019; Gardiner et al., 2018; Klironomos et al., 2013; Pimpinelli & Piacentini, 2020). As such, as our models of RI grow in nuance to match the complexity of natural populations, the incorporation of mechanisms such as epigenetics will become essential. Due to the diversity of fidelity, genomic scales, geographic scales and time scales over which epigenetic marks operate, they have the potential to exhibit a vast array of influences on local adaptation and RI. Even if we find that epigenetics rarely affects RI in natural populations, it is worthwhile to understand why natural circumstances tend to eliminate epigenetic barriers to gene flow. By rooting epigenetic modifications within the framework of genetic variation in both empirical and theoretical work, we can bridge the gap between contemporary organismal processes and speciation.

## AUTHOR CONTRIBUTIONS

All authors contributed to the development of the paper's conceptual framework. NPP wrote the manuscript, and CFC, JF, ZG and PN edited the manuscript.

## CONFLICT OF INTEREST

The authors have no conflicts of interest to declare.

### PEER REVIEW

The peer review history for this article is available at https://publons.com/publon/10.1111/jeb.14033.

## Supporting information


Table S1

## Data Availability

The authors have no raw data to report. R code used to generate Figure 3 is available at https://github.com/planidin/Westram_comment.
