# Supplementary material for: Epigenetics and reproductive isolation: a commentary on Westram et al., 2022
Source: J Evol Biol. 2022 Sep 5;35(9):1188–94. doi: 10.1111/jeb.14033 (PMC9541925; doi:10.1111/jeb.14033)
Supplement: Supplementary file 1 — Table S1 [file JEB-35-1188-s001.docx]

Supplementary Table 1. A list of potential parameters to incorporate into future modeling of epigenetic reproductive isolation (RI) and our predictions for their repercussions.

| **Future modeling directions** | **Predictions** |
| --- | --- |
| Post-migratory induction | Reduces geography-like RI |
| Within generation erasure | Reduces RI and causes greater heterogeneity of epigenetic marks within populations |
| Comparing the relative fitness effects of genetic and epigenetic loci | Under certain circumstances, epigenetic loci may exhibit a greater difference in fitness between habitats |
| Violating assumption of s << m | Epigenetic RI is more robust against the violation of this assumption than genetic RI |
| The environmental gradient context | Clines of RI produced by epigenetic marks will generally be less steep than genetic clines |
| Interactions among > 2 demes | Outcome is highly dependent on the relative fitness of epigenetic states among habitats |
| Weak induction of epigenetic marks | Reduces RI and causes greater heterogeneity of epigenetic marks within populations |
| Alignment of fitness effects among multiple loci | Potentially strong effects of alignment on RI. Influences many mechanisms *e.g.,* genetic loci, epigenetic loci, and geographic barriers |
| Dynamics specific to certain epigenetic marks | Highly context specific *e.g.,* temporal dynamics of siRNAs |
